# Quinine induced simvastatin toxicity through cytochrome inhibition - a case report

**DOI:** 10.1186/s12877-016-0337-8

**Published:** 2016-10-01

**Authors:** Johannes M. Just, Klaus Weckbecker, Katja S. Just

**Affiliations:** 1 Institute of General Practice and Family Medicine Bonn, University Clinic Bonn, Siegmund-Freud Str. 25, 53127 Bonn, Germany; 2 Research Division, Federal Institute for Drugs and Medical Devices, Bonn, Germany

**Keywords:** Geriatrics, Adverse drug event, Adverse drug reaction, Aged 80, and over, Drug interaction, Cytochrome P-450 Enzyme System

## Abstract

**Background:**

Nocturnal leg cramps are painful, involuntary muscle contractions commonly seen in elderly. While mostly harmless, they can severely impair quality of life and often disrupt sleep. Adverse drug effects may be responsible for a fraction of nocturnal leg cramps but often go unrecognized, resulting in additional prescribing intended to deal with adverse effects that might be better addressed by reduction, substitution, or discontinuation of the offending agent.

**Case presentation:**

An 87 year old female presented as outpatient in family medicine with nocturnal leg cramps which had been present for five years and increasingly burdened her quality of life. She had been using quinine 200 mg once daily for symptomatic relief but the cramps kept returning with increasing intensity. During clinical examination we found neither structural nor neurological or metabolic disorders that explained her symptoms. When doing a medication analysis, we found that she was taking a statin together with quinine. Quinine is a cytochrome P450 isoenzyme 3A4 inhibitor, the very enzyme which is involved in the metabolism of most statins. Therefore the use of both substances simultaneously increases blood levels of the statin thereby increasing the risk of side effects including symptomatic myopathy and myalgia. After discontinuing both medications, the patient was, and remained, symptom free.

**Conclusion:**

This case report describes a possible medication interaction that has rarely been noted in literature.

## Background

Nocturnal leg cramps are painful involuntary muscle contractions, typically in the legs or feet. They occur during prolonged rest and often disrupt sleep. They are common among older persons and some individuals experience multiple painful awakenings per night [1, 2].

In a study group of 365 patients with a mean age of 78.5 years, the prevalence of leg cramps was 50 %. Of these 50 %, a fifth had had cramps for longer than 10 years plus two fifth never reported them to their practitioner [1]. Already decades ago, quinine has been suggested with beneficial effects in nocturnal leg cramps [3]. Even though efficacy was reported, data is inconsistent [4] and risk for adverse drug events such as pancytopenia should be considered [5]. In Germany quinine may be used for leg cramps. However, in the US it is not approved for that indication and the U.S. Food and Drug Administration (FDA) as well as the German Federal Institute for Drugs and Medical Devices published warning statements especially due to the risk of thrombocytopenia [6, 7].

Especially older people are more likely to receive a statin prescription. Between 2011 and 2012, 47.1 % of Americans aged 75 and older used a prescription cholesterol-lowering medication in the past 30 days [8]. Statins are known to potentially induce myopathy which can lead to discontinuation of treatment. Patient and drug-related factors such as age, genetics, comorbidities and metabolism, drug-drug interactions, drug transport respectively, can alter the probability [9]. Statins are metabolized by cytochrome P450 3A4 (CYP3A4) isoenzyme. Drugs inhibiting CYP3A4 can therefore interact with statins such as protease inhibitors, cyclosporine, verapamil, amiodarone, macrolide antibiotics and fibrates, which can increase the probability for adverse drug events (ADEs) as in this case of statin-induced myalgia [10]. A case-report on quinine-induced acute renal failure was published. The renal failure resulted in a combination of hemolytic uremic syndrome and rhabdomyolysis with disseminated intravascular coagulation with concordant use of atorvastatin and quinine [11]. In addition, the drug label of quinine sulfate provided by the FDA recommends a reduced dosage of quinine in renal failure due to decreased clearance. Furthermore, the FDA indicates that quinine is an inhibitor of CYP3A4 and therefore patients receiving co-medication, especially CYP3A4 substrates, should be monitored carefully for increased risk of ADEs [12].

ADEs may be responsible for a fraction of nocturnal leg cramps but often go unrecognized, resulting in additional prescribing intended to deal with adverse effects that might be better addressed by reduction, substitution, or discontinuation of the offending agent [13].

In the case of statin induced myopathy and myalgia, additional prescription of quinine might not only be useless but harmful by elevating statin blood levels through cytochrome induction thereby increasing its harmful side effects [11].

We obtained informed consent from the patient. This case report was structured using the CARE Statement Guideline on reporting case reports [14]. For a visual representation see “timeline”.

## Case presentation

### Information

This 87 year old female pensioner presented for the treatment of recurrent nocturnal leg cramps to our family medicine practice. She reported a history of intense, painful sensations in the leg who were associated with sudden muscle hardness. The symptoms regularly appeared in both calves similarly during the night but never during daytime. They had been occurring regularly for five years with a low to intermediate pain intensity. Since three weeks symptoms had been worsening and the patient now described an “intense pain”. She had been treated with quinine by her former family doctor in the past and therefore began to self-medicate with quinine 200 mg once daily from her own stocks achieving only short term symptomatic relief. She asked the consulting doctor to give her another quinine prescription.

The patient had moved here about a year ago and had been a patient of our practice since. She was in very good general condition (Barthel-ADL Score 100 points) and enjoyed good quality of life despite the leg cramps. There was no history of neurologic disorders, hypoglycaemia, diabetes, alcoholism or hypothyroidism. Information on earlier diagnostics concerning the leg cramps was not available.

Her past medical history and medication is displayed in Table 1.

**Table 1:** Patient’s characteristics

Age	87 years
Symptoms	Nocturnal leg cramps of moderate intensity since years. Since three weeks intensity of symptoms increased despite the use of quinine for symptomatic relief
Medical history	myocardial infarction in 2002 with consecutive coronary artery bypass graft (CABG) arterial hypertension chronic kidney disease III knee replacement left + right.
Medication	aspirin 100 mg 1-0-0 bisoprolol 2,5 mg 1-0-0 ramipril 5 mg 1-0-0 simvastatin 40 mg 0-0-1 vitamin D3 1000 IU 1-0-0 torasemide 5 mg 1-0-0 fentanyl patch 12,5 μg once every 3 days. Self-medication: quinine200 mg once daily

### Clinical findings

We conducted a clinical examination based on uptodate’s guideline on nocturnal leg cramps [15].

The 87 old female patient presented in good physical condition. Inspection and palpation of the musculoskeletal system showed no physical abnormalities like muscle atrophy or hypertrophy, bone abnormalities, muscle tenderness or stiffness. Skin examination of the lower extremities was unremarkable despite scars from knee replacement on both sides. Strong arterial pulses were palpable in all relevant locations. Examination of the sensory as well as motor system yielded no pathological findings.

Structural disorders such as flat feet, genu recurvatum, and the hypermobility syndrome were ruled out.

### Diagnostic assessment

Laboratory testing and other studies are usually not required in diagnosing nocturnal leg cramps [15].

**Fig. 1:**
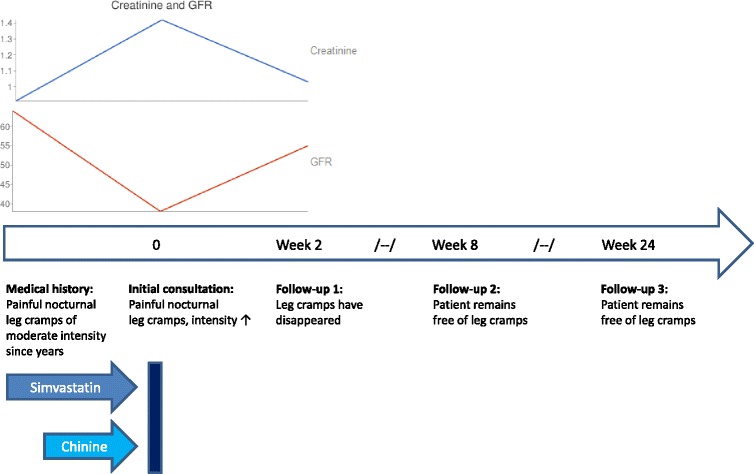
Timeline: no legend

Still we took a blood sample including Creatinine, GFR, Vitamin B12 and folic acid. Renal function had deteriorated compared to prior laboratory findings (Creatinine from 0.91 (range 0.51–0.95) to 1.42, GFR from 64 to 38) (see Fig. 1). The values for tested vitamins were within normal limits. As the blood sample had to be stored for analysis on the following day (outpatient, office setting), we could not analyse creatinine kinase (CK) values. Nocturnal leg cramps are most commonly idiopathic in nature [15]. In this case, past medical history, physical examination and laboratory findings yielded no pathological results. A graphic display is given in the timeline.

In preparing the demanded quinine prescription, we conducted a medication interaction analysis. The analysis showed a warning for the combination of quinine and HMG-CoA reductase inhibitors. Quinine is a CYP3A4 inhibitor, the very enzyme which is involved in the metabolism of most statins [16]. Thereby it may increase blood levels of simvastatin by reducing its first-pass metabolism. One case report documents renal failure, rhabdomyolysis, haemolytic uremic syndrome and disseminated intravascular coagulation after ingestion of quinine along with atorvastatin [11].

Cramps qualify as symptomatic myopathy which is associated with statin therapy and is dose, blood level and cytochrome metabolism dependent [10, 17]. Therefore we considered the combination of the statin and quinine or the statin alone as the most likely cause of the nocturnal leg cramps.

### Therapeutic intervention

Based on these reports we discussed the possible medication interaction with the patient and gave her a resume of the positive effects of the statin. Shared decision making led to an immediate discontinuation of simvastatin and quinine.

### Follow up and outcomes

We reviewed the patient two and three weeks after the discontinuation of her medication. After three weeks she reported to having been “cramp free” for one week for the first time in years, while renal function was improved (Creatinine 1.03, GFR 55) (see Fig. 1). We scheduled a consultation two months and six months later in which the patient continued to be without cramps.

## Discussion

This report shows a case of leg cramps as a possible result of both unnoticed medication side effects as well as medication interaction through cytochrome inhibition.

The discontinuation of both quinine and simvastatin led to a complete and sustained remission of symptoms. Furthermore, renal function improved. With respect to the patient’s age and strain we abstained confirming the diagnosis of a drug-drug interaction by using a challenge-dechallenge-rechallenge testing protocol which is a limitation to this study. In addition we did not want to re-administer quinine which is not first-line therapy in nocturnal leg cramps and is not licensed for the use in leg cramps in the US. Unfortunately simvastatin and CK levels could not be obtained during initial blood sampling due to limitations in laboratory analysis which is a limitation to this study.

Discontinuing the lipid-lowering therapy may lead to an increase in mortality [18]. One observational study found that statin use was independently associated with a reduction in the risk of all-cause mortality (adjusted hazard ratio 0.89; 95 % CI 0.81–0.98; *p* = 0.02) [19]. Therefore, despite limited high-quality evidence, class I recommendations have been made that all patients undergoing CABG should receive statin therapy unless contraindicated [20]. On the other hand, in patients who develop intolerable muscle symptoms statin treatment can be stopped [21]. As the patient already reached old age with overall good quality of life which was greatly impaired by the nocturnal leg cramps, discussing the case with the patient, she chose to discontinue statin therapy.

Thereby, adhered to the Good Palliative–Geriatric Practice algorithm, to assess the possibility to discontinue medication in the elderly in accordance with a 4- step medication decision making model [22, 23]. Following our patient’s decisions, we were not able to proof the diagnosis of a drug-drug interaction by using a challenge-dechallenge-rechallenge testing protocol which is another limitation to this study. A simple side effect of simvastatin without cytochrome related medication interaction is an alternative explanation for this case.

## Conclusion

This case report should remind physicians first, that symptomatic myopathy induced by statins may present with cramp like symptoms and second, symptomatic myopathy is dose related with quinine and other CYP3A4 inhibitors having the potential to increase statin blood levels and thereby symptom intensity.

## Abbreviations

ADEs: Adverse drug events
CABG: Coronary artery bypass graft
CYP: Cytochrome P450
FDA: Food and Drug Administration

## References

[1] Abdulla AJ, Jones PW, Pearce VR (1999). Leg cramps in the elderly: prevalence, drug and disease associations. Int J Clin Pract.

[2] Naylor JR, Young JB (1994). A general population survey of rest cramps. Age Ageing.

[3] Moss HK, Herrmann LG (1940). Use of quinine for relief of "night cramps" in the extremities: Preliminary report. JAMA.

[4] Man-Son-Hing M, Wells G, Lau A (1998). Quinine for nocturnal leg cramps. J Gen Intern Med.

[5] Man-Son-Hing M, Wells G (1995). Meta-analysis of efficacy of quinine for treatment of nocturnal leg cramps in elderly people. BMJ (Clinical research ed).

[6] FDA Drug Safety Communication. New risk management plan and patient Medication Guide for Qualaquin (quinine sulfate). [http://www.fda.gov/Drugs/DrugSafety/PostmarketDrugSafetyInformationforPatientsandProviders/ucm218202.htm]

[7] Chinin gegen nächtliche Wadenkrämpfe (Limptar® N). Bescheid des BfArM zu Änderungen der Produktinformation, einschließlich Einschränkung der Indikation, u.a. wegen des Risikos für schwere Blutbildveränderungen (Thrombozytopenien) im Rahmen eines nationalen Stufenplanverfahrens. [http://www.bfarm.de/SharedDocs/Risikoinformationen/Pharmakovigilanz/DE/RV_STP/a-f/chinin-stp.html]

[8] Prescription Cholesterol-lowering Medication Use in Adults Aged 40 and Over: United States, 2003–2012. [http://www.cdc.gov/nchs/data/databriefs/db177.pdf] 25536410

[9] Abd TT, Jacobson TA (2011). Statin-induced myopathy: a review and update. Expert Opin Drug Saf.

[10] Joy TR, Hegele RA (2009). Narrative review: statin-related myopathy. Ann Intern Med.

[11] Lim AK, Ho L, Levidiotis V (2006). Quinine-induced renal failure as a result of rhabdomyolysis, haemolytic uraemic syndrome and disseminated intravascular coagulation. Intern Med J.

[12] Highlights of prescribing information: Qualanin®. [http://www.accessdata.fda.gov/drugsatfda_docs/label/2014/021799s024lbl.pdf]. Accessed 3 Mar 2016.

[13] Rochon PA, Gurwitz JH (1997). Optimising drug treatment for elderly people: the prescribing cascade. BMJ (Clinical research ed).

[14] Gagnier J, Kienle G, Altman DG, Moher D, Sox H, Riley DS (2013). The CARE guidelines: consensus-based clinical case report guideline development. J Clin Epidemiol.

[15] Nocturnal leg cramps. [http://www.uptodate.com/contents/nocturnal-leg-cramps]. Accessed 3 Mar 2016.

[16] Michalets EL (1998). Update: clinically significant cytochrome P-450 drug interactions. Pharmacotherapy.

[17] Kasiske BL, Wanner C, O'Neill WC (2006). An assessment of statin safety by nephrologists. Am J Cardiol.

[18] Kulik A, Ruel M (2011). Lipid-lowering therapy and coronary artery bypass graft surgery: what are the benefits?. Curr Opin Cardiol.

[19] Philip F, Blackstone E, Kapadia SR (2015). Impact of statins and beta-blocker therapy on mortality after coronary artery bypass graft surgery. Cardiovascular Diagn. Ther..

[20] McIlroy DR, Myles PS (2015). Does the use of statins improve outcomes in coronary artery bypass graft surgery?. Expert Rev Cardiovasc Ther.

[21] McKenney JM, Davidson MH, Jacobson TA, Guyton JR (2006). Final conclusions and recommendations of the National Lipid Association Statin Safety Assessment Task Force. Am J Cardiol.

[22] Garfinkel D, Mangin D (2010). Feasibility study of a systematic approach for discontinuation of multiple medications in older adults: addressing polypharmacy. Arch Intern Med.

[23] Holmes HM, Hayley DC, Alexander GC, Sachs GA (2006). Reconsidering medication appropriateness for patients late in life. Arch Intern Med.

